# In Vitro Modeling of Mycelium Biomass Growth Kinetics of the Novel Fungicolous Species *Xylaria karsticola* NBIMCC 9097, with Insights into Its Antimicrobial Potential

**DOI:** 10.3390/jof12030177

**Published:** 2026-03-01

**Authors:** Galena Angelova, Zlatka Ganeva, Bogdan Goranov, Nikoleta Kaneva, Mariya Brazkova, Petya Stefanova, Denica Blazheva

**Affiliations:** Department of Microbiology and Biotechnology, University of Food Technologies, 26 Maritsa Blvd., 4002 Plovdiv, Bulgaria; g_angelova@uft-plovdiv.bg (G.A.); z_ganeva@uft-plovdiv.bg (Z.G.); b_goranov@uft-plovdiv.bg (B.G.); nikoletakaneva@yahoo.com (N.K.); petyastefanova@uft-plovdiv.bg (P.S.); d_blazheva@uft-plovdiv.bg (D.B.)

**Keywords:** *Xylaria karsticola*, Bulgarian fungicolous ascomycetes, mycelial growth modeling, antimicrobial activity

## Abstract

*Xylaria karsticola* NBIMCC 9097 is a recently described and rare fungicolous species originating from Bulgaria. Understanding its growth behavior and bioactive potential is essential for evaluating its biotechnological and pharmaceutical relevance. In the presented study, we model the in vitro growth kinetics of *X. karsticola* mycelium under submerged cultivation and assess its antimicrobial activity. Optimization of MCM and MYB media markedly increased biomass yields to 20.11 and 23.25 g/dm^3^, respectively, compared with non-optimized media (9.9 ± 0.21 and 10.8 ± 0.28 g/dm^3^). The maximum specific growth rate was higher in the MCM (0.803 ± 0.004 h^−1^) in comparison with the MYB medium (0.711 ± 0.003 h^−1^); however, the MYB medium supported greater biomass accumulation and more efficient substrate utilization, reflected by a higher utilization coefficient (0.9900 ± 0.001 versus 0.9644 ± 0.005). The antimicrobial activity was evaluated using agar disk diffusion and minimum inhibitory concentration assays against Gram-positive and Gram-negative bacteria and yeasts. Hexane and ethyl acetate extracts were most effective against *Pseudomonas aeruginosa* ATCC 9027 (MIC 0.067 and 0.059 mg/cm^3^), while notable anti-yeast activity was observed, particularly against *Wickerhamomyces anomalus*, *Saccharomycodes ludwigii*, and *Pichia membranifaciens*. The lowest MIC (0.02 mg/cm^3^) was recorded for the water biomass extract against *S. ludwigii* indicating potent antimicrobial activity against the tested microorganism. These findings identify *X. karsticola* as a potential source of antimicrobial metabolites and provide a strong motivation for comprehensive metabolomic profiling and systematic optimization of its cultivation.

## 1. Introduction

The demand for innovative and efficacious natural products that can provide therapeutic effect across a wide range of human diseases continues to expand, presenting an ongoing challenge for modern medicine [1,2,3,4]. Nature is increasingly recognized as a significant reservoir of bioactive molecules, essential for the discovery and development of new pharmaceuticals targeting a variety of disorders. As the global threat of antibiotic resistance continues to escalate, the pursuit of new antimicrobial agents has become increasingly urgent [5,6,7].

Fungi, in particular, represent a vast yet largely untapped reservoir of biologically active compounds with significant potential for pharmaceutical and nutraceutical development [8]. Despite this, fewer than 10% of all fungal species have been described, and only a small proportion have been systematically investigated for their therapeutic properties [9,10,11,12]. The continuous emergence of multidrug-resistant pathogens has intensified the search for novel antimicrobial agents from unexplored fungal taxa, particularly those occupying specialized ecological niches such as fungicolous species [13,14]. Occupying highly competitive microhabitats, these fungi have evolved intricate biochemical strategies to secure ecological niches, often relying on the production of specialized metabolites with antimicrobial or cytotoxic functions. Such compounds are thought to mediate antagonistic interactions, inhibit competitors, or modulate host physiology, thereby providing a selective advantage [15,16]. Consequently, fungicolous fungi are hypothesized to possess unique biosynthetic pathways that give rise to chemical scaffolds rarely encountered in saprotrophic or endophytic species [17,18]. The production of secondary metabolites in fungi is profoundly influenced by their growth behavior under controlled conditions, which is crucial for optimizing both biomass accumulation and metabolite synthesis. Mycelial biomass serves as a key indicator of fungal growth and metabolic activity, reflecting the organism’s physiological responses to environmental and nutritional factors [19]. Investigating biomass growth dynamics under in vitro conditions enables systematic evaluation of variables that affect fungal metabolism and facilitates the establishment of standardized culture protocols for downstream biotechnological applications [20]. Controlled in vitro cultivation thus provides a robust framework for modulating growth parameters in a reproducible manner, ultimately enhancing the yield and structural diversity of bioactive metabolites [21,22,23,24]. Despite their ecological and evolutionary significance, the chemical and physiological potential of fungicolous fungi remains insufficiently characterized [25,26].

The genus *Xylaria* Hill ex Schrank (*Ascomycota*, *Xylariaceae*) represents a taxonomically and ecologically diverse group of ascomycetous fungi, distinguished by their ability to colonize a wide range of terrestrial and marine habitats and by the presence of both anamorphic and teleomorphic stages in their life cycle [27,28,29,30]. *Xylaria* species are predominantly saprotrophic, decomposing lignocellulosic plant material such as decaying wood, bark, and leaf litter, and thereby playing a crucial role in nutrient cycling and carbon turnover within forest ecosystems [30]. Although current evidence on the occurrence and distribution of *Xylaria* species colonizing mushroom fruiting bodies remains scarce, recent studies have reported the presence of fungicolous *Xylaria* species [31,32]. The interaction between fungi and their hosts are believed to exert strong evolutionary pressures that have driven the diversification of their secondary metabolism [33,34,35].

Owing to the remarkable biochemical and ecological diversity of the *Xylaria* genus, the past few decades have witnessed systematic investigations into its species for their potential in secondary metabolite production [10,36]. Notably, the most significant recent discoveries of bioactive metabolites have predominantly originated from endophytic *Xylaria* species [37,38]. From a chemical standpoint, *Xylaria nigripes*, *Xylaria polymorha*, *Xylaria hypoxylon*, *Xylaria curta*, *Xylaria longipes*, *Xylaria arbuscula*, and *Xylaria primorskensis* are the most reported as a prolific producer of diverse classes of secondary metabolites, including xyloketals, cytochalasins, xylariolides, xanthones, terpenoids, and polyketides, many of which exhibit antimicrobial, cytotoxic, antioxidant, immunosuppressive, anti-inflammatory, neuroprotective, and enzyme-inhibitory activities, rendering them highly attractive for drug discovery and agrochemical development [10,32,39,40,41,42,43,44,45,46,47,48,49]. Despite the increasing recognition of *Xylaria* species as prolific producers of bioactive metabolites, the information on their in vitro growth under controlled culture conditions and the modeling of associated growth parameters remains scarce.

*Xylaria karsticola* NBIMCC 9097 was isolated from the basidiocarp of *Macrolepiota procera* (*Basidiomycota*) collected in the Stara Planina Mountain, Bulgaria, representing the second report of this species in Europe [31]. The ecological origin of this strain suggests the presence of complex biotic interactions that may stimulate the biosynthesis of antimicrobial metabolites as competitive or defensive responses. However, the influence of nutritional parameters on its mycelial growth dynamics has not yet been systematically investigated. Understanding these physiological and ecological aspects is essential for identifying optimal cultivation strategies to maximize mycelial biomass yield—an important potential source of antimicrobial and other bioactive compounds. Such insights could advance both ecological understanding and biotechnological exploitation of fungicolous *Xylaria* species.

In light of these considerations, the present study focuses on *X. karsticola* NBIMCC 9097, a fungicolous isolate maintained in the National Bank for Industrial Microorganisms and Cell Cultures of Bulgaria (NBIMCC, Bulgaria). The primary objectives were to establish reproducible in vitro submerged cultivation in different nutrients, to evaluate the effects of nutritional modeling on the mycelial growth dynamics, and to provide insight into the antimicrobial potential of the strain.

## 2. Materials and Methods

### 2.1. Fungal Strain

The ascomycete strain *X. karsticola* is part of the microbial collection of the Department of Microbiology and Biotechnology, University of Food Technologies, Plovdiv, Bulgaria. This strain was previously isolated, molecularly identified, and deposited in the National Bank for Industrial Microorganisms and Cell Cultures under accession number 9097. The culture was grown on Mushroom Complete Medium (MCM) comprising 20.0 g/dm^3^ glucose, 0.5 g/dm^3^ KH_2_PO_4_, 1.0 g/dm^3^ K_2_HPO_4_, 0.5 g/dm^3^ MgSO_4_·H_2_O, 2.0 g/dm^3^ peptone, 2.0 g/dm^3^ yeast extract, and 2.0 g/dm^3^ agar, with a pH of 5.5 prior to sterilization, and incubated at 25 °C for 7 days. The full-grown culture was maintained on the same medium at 4 °C, and subculturing onto fresh medium was performed every 90 days.

### 2.2. In Vitro Cultivation Procedures

#### 2.2.1. Screening of Carbon Sources

The effect of different carbon sources and nutrient medium on the mycelial growth of *X. karsticola* was evaluated under in vitro conditions. To screen carbon sources, *X. karsticola* was cultivated on MCM agar in which glucose was individually substituted with fructose, sucrose, maltose, raffinose, arabinose, cellobiose, or cellulose at a concentration of 20 g/dm^3^. The sterilized media were cooled to 45 °C and poured into 90 mm Petri dishes, which were inoculated with 10 mm agar discs from a 7-day-old culture, placed in the center of the Petri dishes and incubated at 25 ± 1 °C. Mycelial growth was assessed by measuring colony diameter at regular intervals and by visual assessment of colony density until full agar coverage. All treatments were conducted in triplicate (n = 3), and the mean values were used for analysis.

#### 2.2.2. Screening of Nutrient Media and Composition Optimization

To evaluate the effect of nutrient media on mycelial biomass production, *X. karsticola* was cultivated under submerged conditions in five liquid media (Table 1). Cultivations were performed in 500 cm^3^ Erlenmeyer flasks containing 100 cm^3^ of medium. The flasks were inoculated with vegetative biomass obtained from a 7-day-old single MCM slant culture and incubated at 25 °C, with agitation at 220 rpm for 7 days in the dark. Mycelial biomass was harvested by filtration and quantified as dry weight (g/dm^3^).

Optimization of medium composition for enhanced mycelial biomass production was conducted using a statistically designed experimental approach. An orthogonal central composite design (CCD; 2^3^) with axial (star) points and block structure was employed. The axial distance (α) was determined based on the number of center-point replications. The range and the levels of the variables investigated in this study are given in Section 3.2.

After identifying the significant influencing factors and constructing the experimental design, the initial variables were coded using the following relationship:

(1) $$x_{i}=\frac{\left(Z_{i}-Z_{i}^{0}\right)}{\Delta Z_{i}}$$

where x_i_ is the coded value of the variable Z_i_; Z_i__0_ is the natural (actual) value of the variable at the center point of the design; and ΔZ_i_ is the variation interval of the i-variable. The actual values of the factors at the axial (star) points were determined using Equation (2).

To ensure the orthogonality of the experimental design matrix, the algorithm of Statgraphics Centurion XVIII^®^ (Version 18.1.16) performed a linear transformation of the quadratic terms in the experimental matrix:

(2) $$X_{j}=X_{j}^{2}-{\;\bar{X}}_{j}^{2}=X_{j}^{2}-\frac{\sum_{i=2}^{N}X_{\mathrm{ij}}^{2}}{N}$$

where N is the number of experiments.

All remaining transformations, as well as the determination of the main statistical parameters used to evaluate the influence of individual factors and the adequacy of the mathematical model, were incorporated into the algorithm of Statgraphics Centurion XVIII^®^.

The dynamics of mycelial biomass accumulation and the estimation of the growth kinetic parameters were performed by cultivation of *X. karsticola* in the optimized media for 15 days, where the mycelial biomass was harvested by filtration and quantified as dry weight (g/dm^3^) in 48 h intervals.

#### 2.2.3. Modeling of the Mycelium Growth Kinetics

For the modeling of the growth kinetics, two approaches were employed: the logistic growth model (Equation (3)) and the reversible autocatalytic growth model (Equation (4)) [50,51,52].

(3) $$\frac{\mathrm{dX}}{d\tau }=\mu_{\max}.X-\beta .X^{2}\Rightarrow \frac{\mathrm{dX}}{d\tau }=\mu_{\max}.X-\frac{\mu_{\max}}{X_{\max}}X^{2}\Rightarrow X=\frac{X_{0}.X_{\max}}{X_{0}+\left(X_{\max}-X_{0}\right)e^{-\mu_{\max}\tau }}$$

(4) $$\frac{\mathrm{dX}}{d\tau }=k_{1}S_{0}^{\prime }X-\frac{S_{0}k_{1}}{X_{\max}}X^{2}\Rightarrow X=\frac{X_{0}S_{0}^{\prime }\frac{K}{1+K}}{X_{0}-\left(S_{0}^{\prime }\frac{K}{1+K}-X_{0}\right)e^{-k_{1}S_{0}^{\prime }\tau }}\;X_{\max}=S_{0}^{\prime }\frac{K}{1+K}$$

where µ_max_ is the maximum specific growth rate (d^−1^); X_0_, X, and X_max_ are the initial, current, and maximum biomass concentrations (g/dm^3^); β is the intrapopulation competition coefficient (g/dm^3^·d); k_1_ is the biomass formation rate constant (d^−1^); S_0_′ is the initial substrate amount in cell units (g/dm^3^); K/(1 + K) represents the substrate utilization coefficient; and τ is the cultivation time (days).

### 2.3. Assessment of the Antibacterial Potential

#### 2.3.1. Submerged Cultivation of *X. karsticola* in the Optimized Medium

The submerged cultivation was conducted in 500 cm^3^ Erlenmeyer’s flasks containing 100 cm^3^ nutrient medium with optimized composition. The flasks were placed on a rotary shaker at 220 rpm and 25 °C for 9 days. At the end of the cultivation process the mycelium biomass was harvested by filtration, washed with distilled water, lyophilized, and then ground into a fine powder and used for extract preparation.

#### 2.3.2. Preparation of Extracts from Mycelial Biomass and Cultural Liquid

Extracts from the mycelial biomass were prepared according to Stefanova et al. [53]. The solvents used for the extract preparation were of analytical grade unless otherwise stated. The lyophilized mycelial biomass (0.5 ± 0.05 g) was combined with 20 mL of each solvent—water, methanol, ethanol, butanol, ethyl acetate, methylene chloride, or hexane. For the cultural liquid—methylene chloride, buthanol, hexane, and ethyl acetate were used in a 1:1 ratio. The mixtures were shaken at 150 rpm for 24 h at 25 °C, followed by centrifugation at 6000 rpm for 15 min at 4 °C, and the resulting supernatants were collected and stored at −18 °C. A second extraction was carried out under identical conditions by adding fresh solvent to the remaining biomass/cultural liquid, followed by centrifugation and collection of the new supernatants. All extractions were performed in triplicate. The obtained extracts from each extraction were combined and concentrated to dryness under vacuum at 40 °C. The dried residues were then reconstituted in dimethyl sulfoxide (DMSO) to achieve a final concentration of 10 mg dry weight/mL and sterilized by filtration through 0.45 µm sterile PTFE syringe filters (Merck, Darmstadt, Germany).

#### 2.3.3. Determination of the Antimicrobial Activity of the *X. karsticola* Extracts

In the antimicrobial assays, the following strains from the collection of the Department of Microbiology and Biotechnology at the University of Food Technologies, Plovdiv, Bulgaria were used: *Escherichia coli* ATCC 8739, *Eterococcus faecalis* ATCC 19433, *Salmonella enterica* ssp. *enterica* ser. Enetritidis ATCC 13076, *Staphylococcus aureus* ATCC 25923, *Pseudomonas aeruginosa* ATCC 9027, *Listeria monocytegenes* ATCC 8787, *Proteus vulgaris* G, *Klebsiella pneumonia* ATCC 13883, *Bacillus subtilis* ATCC 6633, *Bacillus cereus* ATCC 11778, *Wickerhamomyces anomalus*, *Rhodotorula mucilaginosa*, *Saccharomyces cerevisiae*, *Saccharomycodes ludwigii*, and *Pichia membranifaciens*. The bacterial strains and *C. albicans* were maintained on LBG agar with the following composition (g/dm^3^): peptone from casein—10.0; yeast extract—5.0; glucose—10.0; NaCl—10.0; agar—15.0; and a pH of 7.0 prior to sterilization. The yeast strains were maintained on Wort agar.

The disk diffusion assay procedure involved spreading a suspension of each test microorganism (10^6^ CFU/cm^3^) onto specific nutrient agar media (LBG for both the bacteria and *C. albicans* and Wort agar for the rest of the yeasts). On the surface of the nutrient medium, paper disks soaked in the tested extracts were placed. The Petri dishes were then incubated at appropriate temperatures (37 °C for bacteria and *C. albicans*, and 30 °C for the rest of the yeasts) for 24–48 h. After incubation, the inhibition zones around each disk were measured, with zones larger than 6 mm considered zones of inhibition. Each test was performed in triplicate, and the results were reported as mean values of the inhibition zone diameters.

The minimum inhibitory concentration for each extract was evaluated according to the CLSI methods M07-A9 and M27-A3 [54,55]. The extracts were subjected to serial two-fold dilutions in Mueller–Hinton broth (Merck, Germany) for the bacteria and RPMI-1640 medium for the yeasts using a 96-well microtiter plates. Then, each well was inoculated with a bacterial suspension with concentration of 5 × 10^5^ CFU/cm^3^ or yeast suspension with a concentration of 5 × 10^3^ CFU/cm^3^. After mixing, the plates were incubated at 37 °C for 18 h for the bacteria and *C. albicans* and 30 °C for 24 h for the rest of the yeasts. The MIC was the lowest concentration of extract which completely inhibited the growth of the test microorganism.

### 2.4. Statistical Analysis

All the experiments were conducted in triplicate (n = 3), and the values were expressed as mean values ± SD. The statistical significance was determined by the analysis of variance (ANOVA and Tukey’s test and Games -Howell test); the value of *p* < 0.05 indicated a statistical difference.

## 3. Results and Discussion

The subject of this study, *X. karsticola*, was isolated from the basidiocarp of *Macrolepiota procera* (*Basidiomycota*) collected in the Stara Planina Mountain region of Bulgaria, representing only the second documented occurrence of this species in Europe [31]. Although species of the genus *Xylaria* are in the focus of scientific interest due to the production of diverse array of bioactive secondary metabolites, to date, no studies have examined the kinetics of the mycelium growth, the metabolic potential nor the biological activity of *X. karsticola* biomass obtained by in vitro submerged cultivation.

### 3.1. Screening of Carbon Sources

Since *X. karsticola* is a newly identified species no in vitro physiological, nutritional, or cultivation data have been reported to date. Consequently, its growth requirements under controlled conditions have remained entirely unknown. The applied screening approach enabled the identification of nutrient components that significantly promote biomass production and establishes a foundational framework for future optimization, kinetic analyses, and biotechnological investigations of this species.

**Figure 1:**
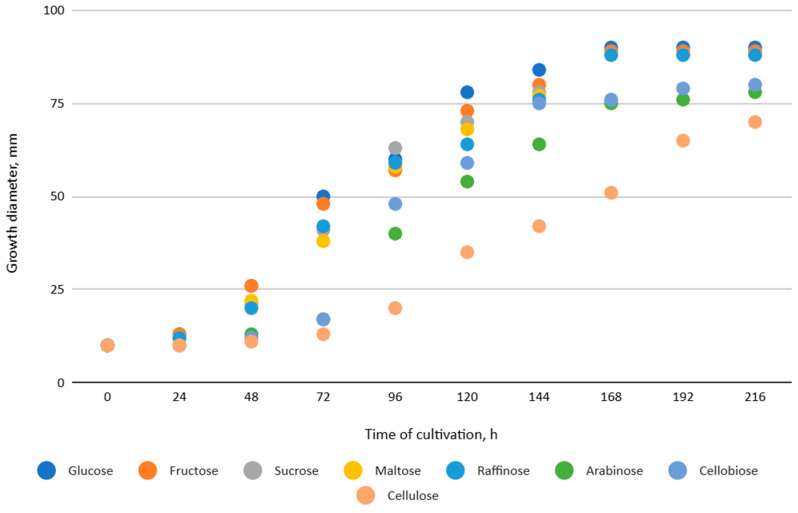
Mycelial growth of *X. karsticola* on different carbon sources.

*Xylaria karsticola* grew on all tested carbon sources, demonstrating broad metabolic versatility (Figure 1 and Table 2A,B). The most rapid and robust mycelial development occurred on glucose, fructose, sucrose, maltose, and raffinose.

Substrate mycelium expanded radially from the colony center, forming interwoven structures, while aerial mycelium appeared white and velvety within 24–48 h (Table 2A). The culture showed the highest initial growth rates, with colony diameters doubling to 20 mm within 48 h, followed by sustained expansion and almost full Petri dish coverage within 144 h. Glucose supported central pigmentation in later stages, whereas fructose produced similar growth without pigment. Maltose, sucrose and raffinose promoted rapid, uniform expansion, peaking between 96 and 120 h, during which the colony expanded by approximately 4 mm per day, followed by gradual decreases in mycelial density, with an average radial increase of about 2 mm per day, likely due to depletion of the available substrate. Differences among these carbon substrates were primarily in mycelial density, and pigment formation. Our observations suggest that pigment formation in *X. karsticola* reflects the physiological maturity of the culture. The use of pigment-producing cultures appears to promote faster adaptation to new growth conditions, as evidenced by a shortened lag phase and accelerated subsequent growth. This indicates that pigment production may be associated with metabolic readiness and enhanced developmental capacity in *X. karsticola*.

Cultivation of *X. karsticola* on arabinose—and cellobiose-containing medium (Table 2B)—demonstrated moderate growth, and even after 216 h there was no full Petri dish coverage. When arabinose was used, the colony diameter increased by only 7 mm after 72 h. The mycelium displayed a wavy morphology, with colonies forming raised concentric margins resembling a floral pattern. These colonies exhibited an atypical structure and did not produce pigment. *X. karsticola* grew moderate on cellobiose, indicating slower efficiency in utilizing this disaccharide compared to simpler sugars. Nevertheless, the fungus formed dense, white mycelium, suggesting active biomass accumulation despite reduced radial expansion. Colony growth plateaued after 192 h, likely reflecting nutrient limitation. Although *X. karsticola* grows robustly on woody substrates in its natural habitat, growth on cellulose as the sole carbon source was limited. Aerial mycelium was sparse, and colony diameter doubled only after 96 h. This likely reflects the slow enzymatic hydrolysis of cellulose and the absence of complementary nutrients present in wood that support rapid mycelial development.

The relatively rapid assimilation of glucose, fructose, saccharose, and maltose indicates a flexible carbon metabolism that could be advantageous for cultivation and different biotechnological applications. The type of carbon source influences not only mycelial biomass accumulation but also the metabolic profile and yield of bioactive secondary metabolites produced by the fungus. Liu et al. [56] applied the one-factor-at-a-time method and evaluated the mycelial biomass growth of *Xylaria striata* by measuring mycelial dry weight. Their results indicated that maltose, glucose, or corn powder served as optimal carbon sources, which is consistent with our findings. In this study, glucose was selected as the primary carbon source, providing a baseline for growth of *X. karsticola* and its metabolic evaluation. Future investigations will examine the effects of alternative carbon sources on mycelial development and metabolite production.

### 3.2. Screening of Nutrient Media and Composition Optimization

Cultivation optimization is a key strategy to enhance both mycelial biomass and metabolite production. Given the critical role of nutrient composition, five media formulations with glucose as carbon source were evaluated for their ability to support mycelial growth under submerged cultivation, and the results are presented in Table 3.

As shown in Table 3, mycelial biomass production varied among the tested media, with the highest values observed on MYB (10.8 g/dm^3^) and MCM (9.9 g/dm^3^). In contrast, PDB, MEB, and CDB supported comparatively lower biomass accumulation. These findings suggest that media containing complex nutrient components, particularly yeast extract, malt extract and peptone, may better support the growth of *X. karsticola*.

As mentioned above, the nutritional preferences of *X. karsticola* have not been previously investigated, which complicates direct comparison with our findings. Studies on other species within the genus *Xylaria* are also limited; however, several investigations have been reported. Prior to exploring *Xylaria papulis* as a potential and sustainable source of bioactive compounds with high functional activity, López et al. [57] first investigated the optimal liquid media and physical culture conditions for mycelial growth of endophytic fungi *Xylaria* sp. BCC 1067. Among the media evaluated—potato sucrose broth, corn meal broth, rice bran broth, and coconut water—the highest mycelial biomass was obtained in potato sucrose broth. Similarly, Jayasekara et al. [58] reported that yeast extract sucrose medium supported higher biomass production, whereas the highest biological activity was observed in a malt extract peptone medium, highlighting the influence of culture medium composition on both growth and functional performance. Liquid fermentation of *Xylaria longipes* on PDB and glucose peptone yeast resulted in different secondary metabolites anti-psoriasis potential [32].

Overall, our results provide preliminary insights into the nutritional requirements and growth behavior of *X. karsticola* and may serve as a useful reference for future studies on this species and other related *Xylaria* taxa. Based on their superior performance, MYB and MCM were selected for subsequent optimization experiments aimed at refining nutrient composition and improving biomass yield.

The composition of the MCM and MYB media was optimized using an orthogonal central composite design of the 2^3^ type, with star points and a block structure. The star arm length was set at ±α = 1.28719. The response variable selected was the amount of absolutely dry biomass, expressed in g/dm^3^.

The region containing the optimum of the regression model was defined as quasi-stationary and is characterized by pronounced nonlinearity:

(5) $$Y=b_{0}+\sum_{i=1}^{k}b_{i}X_{i}+\sum_{1<j<i<k}^{k}b_{ij}X_{i}X_{j}+\sum_{i=1}^{n}b_{ii}X_{i}^{2}$$

A single-factor experiments were conducted to assess the effects of independent variables (factors), and the corresponding variation ranges were determined. The defined ranges for each medium are presented in Table 4 and Table 5.

The experimental design for the two selected media was generated using Statgraphics Centurion XVIII^®^ software (Version 18.1.16) and are presented in Table 6 and Table 7.

**Figure 2:**
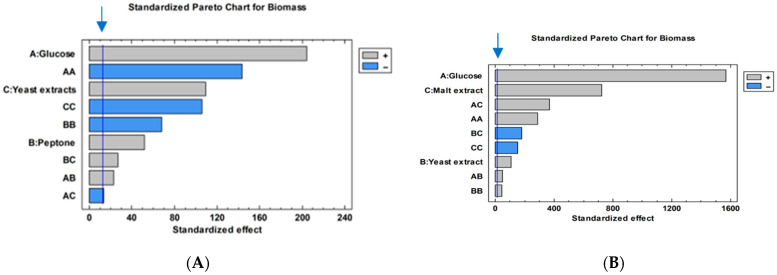
Pareto chart for submerged biomass production by *X. karsticola* in MCM (**A**) and MYB (**B**): positive estimated effects (gray bars); negative estimated effects (blue bars).

A Pareto chart was constructed to evaluate the statistical significance and effects of independent factors of MCM and MYB on *X. karsticola* biomass yield; it also showed the interaction strength between each independent variable (Figure 2). According to the Pareto chart (Figure 2A), glucose, yeast extract, and peptone demonstrate a statistically significant positive effect on the development of *X. karsticola* when cultivated in MCM.

A positive effect on fungal biomass accumulation was also observed for the combined effects of peptone with yeast extract and peptone with glucose. In contrast, the combined effect of glucose and yeast extract was found to be statistically insignificant. As a result of this analysis, the following quadratic mathematical model describing *X. karsticola* biomass yield in MCM was developed:

Biomass = −18.2068 + 0.927325*Glu + 2.68178*P + 5.56438*YE − 0.00974099*Glu^2 + 0.0129453*Glu*P − 0.462471*P^2 + 0.150078*P*YE − 0.722127*YE^2

**Figure 3:**
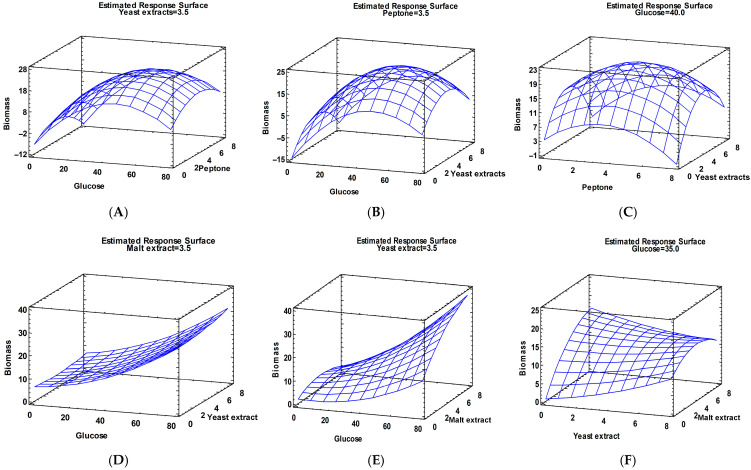
Estimate response surface curves of mycelium biomass from *X. karsticola*, showing the interaction between the concentration of glucose and peptone (**A**), glucose and yeast extract (**B**), and peptone and yeast extract (**C**) in MCM and glucose and yeast extract (**D**), glucose and malt extract (**E**), and yeast extract and malt extract in MYB (**F**).

From the response surface plots (Figure 3A–C) and the derived quadratic model, it is evident that increasing the concentrations of glucose, yeast extract, and peptone leads to a decrease in the biomass yield of *X. karsticola*. This trend is further supported by the negative values of the quadratic coefficients for glucose, peptone, and yeast extract, indicating an inhibitory effect of higher concentrations of these components on biomass production.

An analysis of the Pareto diagram (Figure 2B) revealed that glucose, malt extract, and yeast extract exerted a statistically significant positive effect on the growth of *X. karsticola* in MYB medium. Biomass accumulation of the strain cultivated in MYB medium was likewise positively influenced by the combined effects of glucose with malt extract, glucose with yeast extract, elevated glucose concentration, and yeast extract as an individual factor. In contrast, a negative effect on biomass production was observed for the combined factors yeast extract–malt extract and the doubling of malt extract concentration.

Based on these findings, the quadratic mathematical model describing biomass growth in MYB is expressed as follows:

Biomass = −0.0490127 − 0.0733714*Glu + 0.371431*YE + 2.58111*ME + 0.00270046*Glu^2 + 0.004785*Glu*YE + 0.035765*Glu*ME + 0.0645289*YE^2 − 0.21925*YE*ME − 0.225931*ME^2

**Figure 4:**
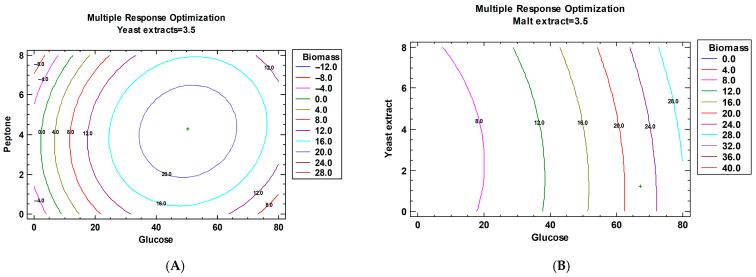
Multiple response optimization—independent variables of MCM (**A**) and independent variables of MYB (**B**).

The estimated response surface charts are presented in Figure 3 and illustrate the combined effects and interactions of the components of MCM and MYB on biomass production, revealing distinct regions associated with maximal responses. These trends were subsequently integrated using multiple response optimization (Figure 4), which identified the optimal combination of factor levels yielding the highest predicted biomass.

The optimized compositions of the MCM and MYB media (Table 8 and Table 9) reveal distinct nutrient requirements for maximal biomass production. In both media, glucose emerged as a key factor influencing fungal growth; however, its optimal concentration differed markedly between the two formulations. In the MCM (Table 8), the optimal glucose concentration (50.46 g/dm^3^) was close to, but not at, the upper limit of the tested range, indicating that high carbon availability enhances biomass accumulation up to a threshold beyond which substrate inhibition is observed. A similar trend is observed for the nitrogen sources. These results indicate that, in MCM, the balance between the carbon and nitrogen sources plays a critical role in biomass production.

In contrast, the optimization of the MYB medium (Table 9) resulted in an optimal glucose concentration corresponding to the upper boundary of the investigated range (67.18 g/dm^3^), suggesting that glucose was the primary growth-limiting factor under these conditions. This result indicates a stronger dependence on carbon supply in MYB compared with MCM.

Differences were also observed in the optimal nitrogen and complex nutrient sources. In the MCM, the optimal concentrations of peptone and yeast extract were intermediate within the tested ranges (approximately 4.30 g/dm^3^), implying that balanced nitrogen supplementation is required to support efficient growth without inhibitory effects. Conversely, in the MYB medium, the optimal yeast extract concentration was relatively low (1.24 g/dm^3^), whereas the malt extract was optimal at its highest tested level (6.07 g/dm^3^). This combination suggests that malt extract may provide additional growth-promoting components, such as vitamins or trace elements, reducing the need for higher yeast extract supplementation.

**Figure 5:**
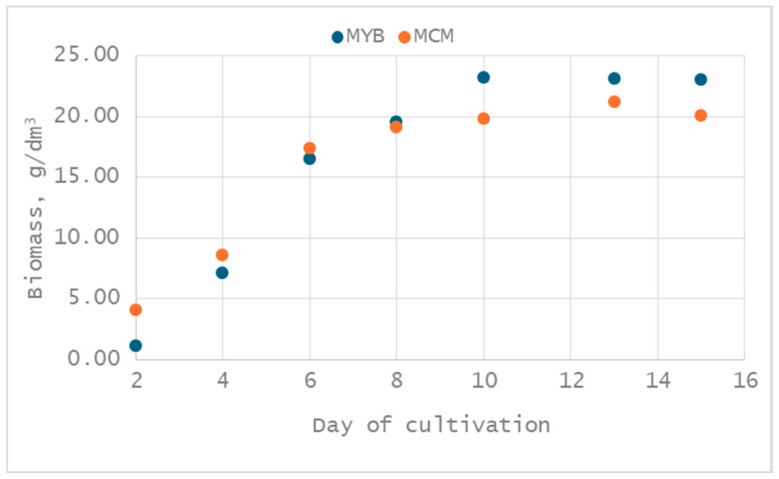
Dynamics of biomass change of *X. karsticola* cultivated in optimized MYB and MCM at 25 °C.

Overall, the optimized media compositions underscore the importance of medium-specific nutrient balancing. While both formulations rely heavily on glucose as the principal carbon source, the contrasting requirements for nitrogen and complex extracts highlight differences in nutrient utilization pathways and growth kinetics, consistent with the observed variations in biomass accumulation between MCM and MYB media. After establishing the optimal composition of the two studied media, a cultivation process was carried out to confirm the optimal composition. The dynamics of biomass change in the two optimized nutrient media were monitored, and the results of the studies conducted are shown in Figure 5.

A comparative analysis of the growth kinetics in MYB and MCM media demonstrates clear differences in both the rate and extent of biomass accumulation. In both media intensive growth was observed from the early stages of cultivation; however, MYB supported a more rapid transition to the stationary phase. In the MYB medium, active biomass accumulation occurred up to day 10, at which point a high biomass concentration of approximately 23.25 g/dm^3^ was achieved and subsequently maintained with minimal variation until the end of cultivation. This rapid attainment of maximal biomass suggests that MYB provides a more readily assimilable nutrient composition, enabling earlier saturation of fungal growth. In contrast, cultivation in the MCM resulted in a more prolonged growth phase, with biomass accumulation continuing until day 13 and reaching a slightly lower maximum concentration of 21.24 g/dm^3^. Although the final biomass concentration decreased marginally to 20.11 g/dm^3^, the extended growth period indicates slower but sustained nutrient utilization in MCM. Consequently, the optimal cultivation time for biomass production in MCM is longer than in MYB, reflecting differences in medium composition and nutrient availability. Importantly, validation experiments confirmed that optimization of both media led to substantial improvements in biomass yield compared with their non-optimized counterparts. The optimized MYB medium resulted in a markedly higher biomass concentration (23.25 g/dm^3^) relative to the non-optimized MYB (10.8 g/dm^3^), while a similar enhancement was observed for MCM, where biomass increased from 9.9 g/dm^3^ in the non-optimized medium to over 20 g/dm^3^ following optimization. Overall, MYB proved to be more efficient for rapid biomass accumulation, whereas MCM supported extended growth with a slightly lower maximum yield, underscoring the importance of medium-specific optimization strategies for fungal cultivation.

Liu et al. [58] employed an orthogonal experimental design and determined that maltose (4%) and soybean powder (0.8%) were the optimal carbon and nitrogen sources that supported the mycelial growth of *X. stirata*. Under those conditions, the highest mycelial biomass was achieved at pH 7, with shaking at 25 °C for 13 days. Culture condition optimization was applied for improving the productivity of 19, 20-Epoxy-cytochalasin Q (B5A) in *Xylaria* sp. sof11 by Zhang et al. [59]. The effects of major medium components, including the carbon and organic nitrogen sources, as well as the concentration of sea salt, were, respectively, investigated through single-factor experiments. As a result, sucrose and fish meal were determined to be the key factors affecting the production of B5A. Response surface analysis confirmed the optimal levels of response variables, which were sucrose, fish meals and filling volume. Eucalyptene A production by *Xylaria* sp. 2508 was optimized using response surface analysis, which identified glucose (2.86% *w*/*v*), peptone (0.78% *w*/*v*) and yeast extract (0.35% *w*/*v*) as the optimal medium components. Under these conditions, eucalyptene A reached a yield of 4.89 × 10^−7^ g/cm^3^ [60].

### 3.3. Modeling of the Mycelium Growth Kinetics

**Figure 6:**
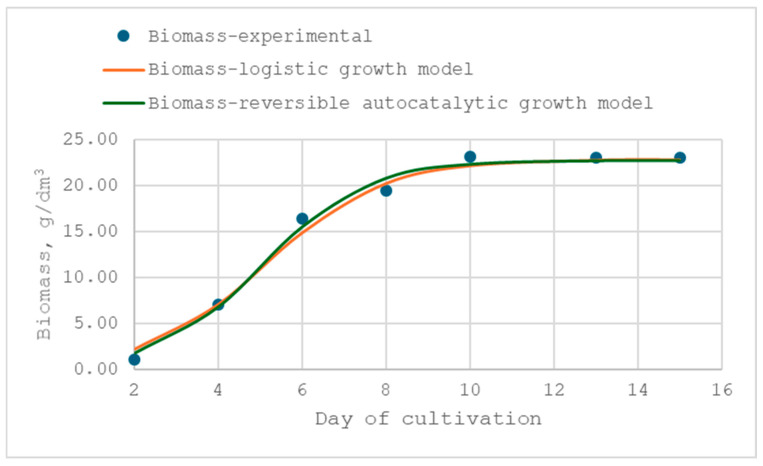
Comparison of experimental biomass concentration data for the *X. karsticola* strain with predictions of the logistic and reversible autocatalytic growth models in MYB medium.

**Figure 7:**
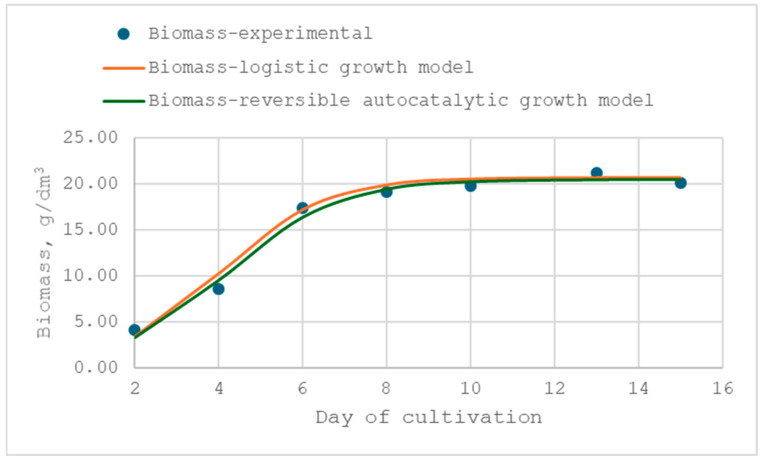
Comparison of experimental biomass concentration data for the *X. karsticola* strain with predictions of the logistic and reversible autocatalytic growth models in MCM.

A comprehensive understanding of process kinetics is crucial for the successful biotechnological application of fungi. The logistic curve and reversible autocatalytic growth models were used to model the kinetics of the growth of *X. karsticola* in submerged cultivation in the previously optimized media. The main observed quantity by which the growth of the fungus was modelled was the biomass. A comparison of the experimental data and those from the models was made, and the results of this comparison could be seen in Figure 6 and Figure 7, and the identified kinetic parameters of the models are presented in Table 10.

The data presented in the figures demonstrate that the applied kinetic models describe the experimental results with high accuracy, as confirmed by the high correlation coefficients. For both media, the coefficients of determination (R^2^) ranged from 0.9868 to 0.9963, indicating an excellent fit between the predicted and experimental values. This confirms the suitability of the selected kinetic model for quantitative characterization of the growth dynamics under the tested conditions. An analysis of the estimated kinetic parameters revealed distinct differences in the growth behavior of *X. karsticola* depending on the cultivation medium. The fungus exhibited a higher maximum specific growth rate in the MCM (µ_max_ = 0.803 ± 0.004 d^−1^) compared with MYB medium (µ_max_ = 0.711 ± 0.003 d^−1^). In contrast, no statistically significant difference was observed for the biomass formation rate constant, with comparable values obtained for MYB (0.0363 ± 0.0012 d^−1^) and MCM (0.0359 ± 0.0004 d^−1^).

A comparison of the two maximum growth rates shows that the absolute difference between the two optimized media is small—Δμ_max_ is 0.091 d^−1^. This corresponds to an approximately 13% increase in µ_max_ in the MCM relative to MYB. This slight acceleration of growth in the initial stages of the process carried out in the MCM does not represent a significant advantage in the industrial cultivation of the strain for the production of biomass and biologically active products. In practical terms, such a difference would result in only a marginal reduction in cultivation time under batch conditions. This conclusion is also confirmed by the close values of the statistically insignificant rate constants of biomass formation, which are 0.0363 ± 0.0012 d^−1^ and 0.0359 ± 0.0004 d^−1^ for MYB and MCM media, respectively. Therefore, although statistically significant, the higher µ_max_ in MCM should be interpreted as biologically measurable but technologically moderate.

The kinetic parameters summarized in Table 10 further indicate that the substrate reserve expressed in cellular units in the optimized media is comparable to the experimentally achieved biomass concentrations. Specifically, the estimated substrate reserve (S′_0_) was higher for the MYB medium (23.05 ± 0.08 g/dm^3^) than for the MCM (21.27 ± 0.04 g/dm^3^), which accounts for the observed differences in final biomass concentrations. The composition of the MYB medium thus provides a larger pool of readily assimilable substrates, supporting higher biomass accumulation relative to MCM. This finding is particularly relevant for applications where final biomass yield, rather than rapid initial growth, is the primary objective.

A statistically significant difference was also observed in the substrate utilization efficiency coefficient (K/(1 + K)), which was higher in the MYB medium (0.9900 ± 0.001) than in the MCM (0.9644 ± 0.005), indicating more efficient substrate conversion into biomass under MYB conditions. Conversely, cultivation in the MCM was associated with a higher coefficient of intra-population competition (β = 0.0389 ± 0.0002 g/dm^3^·d) compared with the MYB medium (β = 0.0311 ± 0.0002 g/dm^3^·d). This suggests stronger competition among cells for available substrates in MCM, likely due to the lower substrate reserve per cellular unit in this medium. Together, these parameters provide mechanistic insight into the observed growth pat-terns and highlight how medium composition influences both growth kinetics and biomass yield.

In conclusion, the kinetic analysis indicates that although *X. karsticola* exhibits a higher maximum specific growth rate in MCM, the MYB medium is more suitable for cultivation aimed at maximizing biomass yield. The higher substrate availability and utilization efficiency in MYB ultimately compensate for the lower growth rate, resulting in superior biomass production compared with MCM. Importantly, the comparative kinetic characterization of the two optimized media provides a rational basis for medium selection depending on the intended biotechnological goal—rapid biomass accumulation versus maximal yield.

As mentioned above, the nutritional preferences of *X. karsticola* have not yet been investigated. Moreover, studies on other species within the genus *Xylaria* are limited, which makes direct comparison of our results challenging. Nevertheless, our findings provide novel baseline data on the nutritional preferences of this species and contribute to a broader understanding of metabolic diversity within the genus. Given the limited kinetic data available for representatives of this genus, the present study establishes quantitative reference parameters that may support future strain improvement, metabolic studies, and process scale-up.

### 3.4. Assessment of the Antibacterial Potential

Although many *Xylaria* species are recognized for producing bioactive secondary metabolites, the antimicrobial potential of *Xylaria karsticola* remains unexplored, motivating this study to evaluate its solvent-extractable compounds against bacteria and yeasts. The antimicrobial activity was evaluated using agar disk diffusion and minimal inhibitory concentration (MIC) assays against a panel of Gram-positive and Gram-negative bacteria, as well as yeasts (Table 11). Extracts were prepared from fungal biomass and culture liquid using solvents with different polarity to assess the contribution of chemically diverse metabolites.

Overall, the antimicrobial activity of *X. karsticola* extracts was selective and strongly dependent on both extraction solvent and target microorganism. The aqueous, methanolic, ethanolic, and butanolic biomass extracts did not exhibit measurable antibacterial effects, suggesting that highly polar constituents play a limited role in antibacterial activity under the tested conditions. A reported disk diffusion screening of methanolic and ethyl acetate extracts from multiple *Xylaria* isolates demonstrated selective antimicrobial activity, with inhibition primarily observed against Gram-positive bacteria and *C. albicans*, while Gram-negative bacteria were largely resistant [39,61]. Among the tested bacteria, the Gram-negative *P. aeruginosa* ATCC 9027 was the most susceptible organism. Several biomass-derived extracts demonstrated measurable inhibition zones, including methanol, butanol, hexane, ethyl acetate, and methylene chloride extracts, whereas culture liquid extracts were largely inactive. Inhibition zones of 8–9 mm, although only slightly larger than the 6 mm disk diameter, were consistently observed in independent replicates (Table 11). These values therefore represent weak but measurable antimicrobial activity rather than experimental variability. Nevertheless, they should be interpreted as low-intensity effects.

MIC data supported these results, with the hexane biomass extract exhibiting the strongest antibacterial activity (MIC = 0.067 mg/cm^3^), followed by the ethyl acetate extract (MIC = 0.59 mg/cm^3^) (Table 12). Certain *Xylaria* strains have been reported to display markedly stronger antibacterial activity, with MIC values in the low microgram per cm^3^ range against *S. aureus* and *Pseudomonas* species, emphasizing the variability in antimicrobial potency across the genus [62,63,64].

Most other Gram-negative bacteria, including *E. coli*, *S. enterica*, *P. vulgaris*, and *K. pneumoniae* were resistant to all tested extracts, with only weak inhibition observed for *E. coli* using methylene chloride biomass extract. Several other authors report lack of antimicrobial activity against *E. coli.* In an investigation of the antimicrobial activity of methanol and ethyl acetate extracts derived from six fungal isolates, including *Xylaria* sp., none of the isolates inhibited the Gram-negative test microorganisms [61]. The same result was observed for the lipophilic and aqueous methanolic crude extracts of *Xyleria feejeensis* [65]. The crude extract of *Xylaria venustula* biomass exhibited MIC of 5 mg/cm^3^, which is comparable to the results for the methylene chloride biomass extract in this study [66].

Moderate antibacterial effects were also observed against the Gram-positive *Bacillus* species. *Bacillus subtilis* ATCC 6633 was inhibited by ethyl acetate biomass extract, corresponding to an MIC of 1.66 mg/cm^3^, while *B. cereus* ATCC 11778 was susceptible to ethyl acetate culture liquid extract, with an MIC of 6.25 mg/cm^3^. Comparable solvent-dependent activity has been described for endophytic *Xylaria* isolates, where ethyl acetate extracts frequently exhibited higher potency than polar extracts against both Gram-positive and Gram-negative bacteria [67].

No inhibitory activity was detected against the rest of the Gram-positive bacteria, including *S. aureus* ATCC 25923 in this study. The only exception is the weak activity of the methylene chloride extracts against *L. monocytogenes.* This contrasts with reports of strong activity against *S. aureus* in some *Xylaria* strains and highlights the strain- and species-specific nature of antimicrobial metabolite production. For example, crude ethyl acetate extracts of wild *Xylaria* sp. strain R005 showed moderate activity against multidrug-resistant *S. aureus* and *P. aeruginosa* (MIC = 120–625 µg/cm^3^) [67]. Canli et al. (2016) reported on inhibition zones of 16 mm against *S. aureus* [68], and Santiago et al. determined an MIC of 2.5 mg/cm^3^ for *Xylaria venustula* against *S. aureus* [66].

Several extracts demonstrated antifungal or anti-yeast activity, particularly against *Candida albicans* ATCC 10231 and non-*Candida* yeasts. Inhibition of *C. albicans* was observed for ethyl acetate, methylene chloride, and butanol extracts, with the MIC value for the butanol culture liquid extract indicating low-to-moderate antifungal potency. Stronger activity was observed against *W. anomalus*, *S. ludwigii*, and *P. membranifaciens*, with particularly low MIC values of the water biomass extract and hexane culture liquid extract against *S. ludwigii* and *P. membranifaciens*, suggesting potent anti-yeast metabolites. The lowest value was observed for the water biomass extract against *S. ludwigii*—0.02 mg/cm^3^. Such yeast-targeted activity has received less attention in prior studies, which have largely focused on filamentous fungi or clinically relevant pathogens. The antifungal activity is consistent with the known ability of *Xylaria* species to produce polyketide-derived secondary metabolites. Chemical studies of *Xylaria* liquid cultures have identified griseofulvin and dechlorogriseofulvin as major antifungal constituents, exhibiting strong activity against multiple phytopathogenic fungi [39]. However, no chemical characterization of the extracts was performed in the present study; therefore, the specific compounds responsible for the observed activity remain to be identified.

*Xylaria* species produce a wide range of structurally diverse secondary metabolites with documented antimicrobial activity. Most bioactive compounds fall into major classes, including polyketides, terpenoids, nitrogen-containing metabolites, lactones, and other small heterocyclic molecules [40]. Polyketides, including griseofulvin-type derivatives, are the best characterized antimicrobial class, showing strong antifungal and moderate antibacterial activity [39,40]. The anti-yeast and moderate antifungal effects observed for *X. karsticola* extracts, particularly in ethyl acetate and methylene chloride fractions, could hypothetically be associated with polyketide-type compounds; however, this assumption requires experimental confirmation. Terpenoids and terpenoid hybrids, frequently reported from *Xylaria*, have demonstrated weak to moderate antibacterial activity [37] and could potentially contribute to the inhibition of *Bacillus* spp. and *P. aeruginosa*. Nitrogen-containing metabolites, lactones, and other small heterocyclic molecules (e.g., pyranones, phthalides) have been reported to exhibit antimicrobial properties and might act additively or synergistically, which could partly explain the selective antimicrobial profile [37,40].

Although comparative data for *Xylaria* species remain limited, previously reported studies indicate considerable variability in antimicrobial potential within the genus. Reported antimicrobial activities range from weak to pronounced effects depending on species, extraction method, and target organism. However, differences in cultivation conditions and assay methodologies complicate direct quantitative comparisons. Within this context, the present study represents the first comprehensive evaluation of *X. karsticola* antimicrobial activity using both disk diffusion and MIC assays. Unlike many previously investigated *Xylaria* species, *X. karsticola* exhibited a distinct profile: moderate antibacterial activity against *P. aeruginosa*, coupled with pronounced anti-yeast activity against *W. anomalus*, *S. ludwigii*, and *P. membranifaciens*. The strong solvent-dependence of activity suggests the involvement of lipophilic secondary metabolites, highlighting *X. karsticola* as a previously unexplored source of bioactive fungal compounds.

## 4. Conclusions

This study provides the first insights into the in vitro nutritional preferences, mycelial growth kinetics and antimicrobial potential of the little-known fungicolous species *Xylaria karsticola* NBIMCC 9097, contributing to a broader understanding of the genus *Xylaria*. Optimization of culture medium composition and cultivation duration revealed that maximum biomass production was achieved using MYB media with an incubation period of 10 days. These findings establish a foundation for efficient mycelial growth and targeted bioactive metabolite production in *X. karsticola*. De novo whole-genome sequencing (WGS), genome annotation, and targeted gene mining in the future are expected to reveal how the genetic potential of this newly identified strain drives the directed biosynthesis of novel metabolites. Further work should focus on bioassay-guided fractionation of the most active solvent extracts to enable the isolation and structural characterization of bioactive compounds. In addition, investigating potential synergistic interactions among distinct metabolite classes may help elucidate the mechanisms underlying the observed selective antibacterial and antifungal activities.

## Figures and Tables

**Table 1:** Composition of the liquid culture media used for submerged cultivation and kinetics modelling of mycelium biomass growth (g/dm^3^).

	MCM	MEB *	MYB *	PDB *	CDB *
Glucose	20.0	-	10	20.0	-
Sucrose	-	-	-	-	30.0
Peptone	2.0	3.0	-	-	-
Yeast extract	2.0	-	5.0	-	-
Malt extract	-	30.0	3.0	-	-
Potato extract	-	-	-	4.0	-
NaNO_3_	-	-	-	-	2.0
MgSO_4_·7H_2_O	0.5	-	-	-	0.5
K_2_HPO_4_	1.0	-	-	-	1.0
KH_2_PO_4_	0.5	-	-	-	-
KCl	-	-	-	-	0.5
FeSO_4_·7H_2_O	-	-	-	-	0.01

* MEB—malt extract broth; MYB—malt extract yeast extract broth; PDB—potato dextrose broth; CDB—Czapek–Dox broth.

**Table 2:** Mycelial growth of *X. karsticola* on different carbon sources (**A**)—24–168 h; (**B**)—48–216 h.

(**A**)	24 h	48 h	72 h	96 h	120 h	144 h	168 h
Glucose	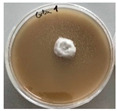	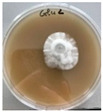	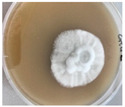	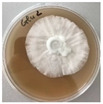	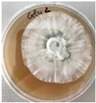	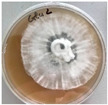	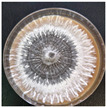
Fructose	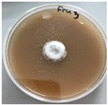	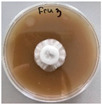	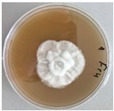	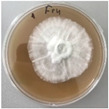	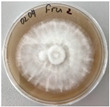	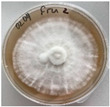	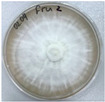
Sucrose	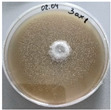	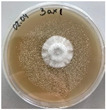	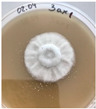	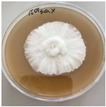	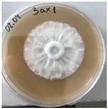	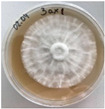	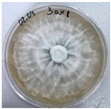
Maltose	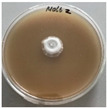	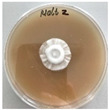	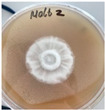	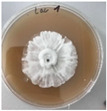	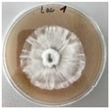	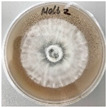	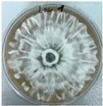
Rafinose	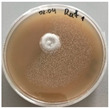	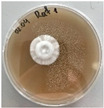	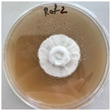	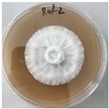	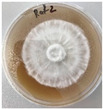	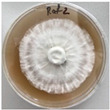	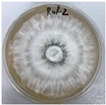
(**B**)	48 h	72 h	96 h	144 h	168 h	192 h	216 h
Arabinose	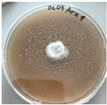	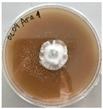	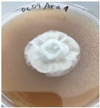	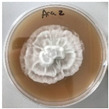	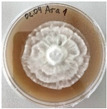	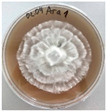	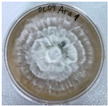
Celobiose	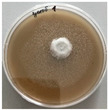	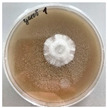	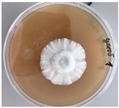	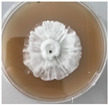	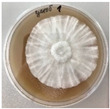	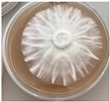	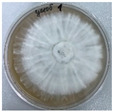
Cellulose	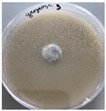	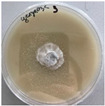	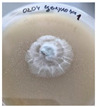	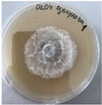	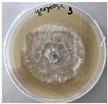	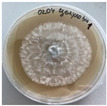	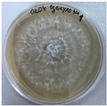

**Table 3:** Biomass yield after submerged cultivation on different media.

	MCM	MEB	MYB	PDB	CDB
Biomass, g/dm^3^	9.9 ± 0.21 ^b^	8.8 ± 0.18 ^c^	10.8 ± 0.28 ^a^	7.8 ± 0.16 ^d^	6.0 ± 0.14 ^e^

Data are expressed as mean ± SD (n = 3); a, b, c, d, e—different letters in the rows indicate significantly different values (Tukey HSD tests, *p* < 0.05).

**Table 4:** Experimental range and levels of the independent variables (g/dm^3^) for the MCM.

Component, g/dm^3^	−α	Lower Level	Basic Level	Higher Level	+α
Glucose	14.26	20	40	60	65.74
Yeast extract	0.93	1.5	3.5	5.5	6.07
Peptone	0.93	1.5	3.5	5.5	6.07

**Table 5:** Experimental range and levels of the independent variables (g/dm^3^) for the MYB.

Component, g/dm^3^	−α	Lower Level	Basic Level	Higher Level	+α
Glucose	2.82	10	35	60	67.18
Yeast extract	0.93	1.5	3.5	5.5	6.07
Malt extract	0.93	1.5	3.5	5.5	6.07

**Table 6:** Experimental design for optimization of the MCM to enhance mycelial biomass production.

Independent Variables			Response
Glucose, g/dm^3^	Peptone, g/dm^3^	Yeast extract, g/dm^3^	Biomass, g/dm^3^
40.0	3.5	3.5	21.04
60.0	5.5	1.5	16.47
40.0	3.5	3.5	20.15
65.74	3.5	3.5	20.04
20.0	1.5	5.5	9.11
40.0	3.5	0.93	11.00
40.0	3.5	6.07	20.52
20.0	5.5	5.5	11.04
40.0	0.93	3.5	17.17
14.26	3.5	3.5	10.11
20.0	5.5	1.5	7.245
60.0	5.5	5.5	19.37
60.0	1.5	5.5	15.07
40.0	6.07	3.5	19.78
20.0	1.5	1.5	7.41
60.0	1.5	1.5	14.87

**Table 7:** Experimental design for optimization of the MYB medium to enhance mycelial biomass production.

Independent Variables			Responses
Glucose, g/dm^3^	Peptone, g/dm^3^	Yeast extract, g/dm^3^	Biomass, g/dm^3^
10.0	5.5	1.5	6.06
60.0	5.5	1.5	17.23
2.82	3.5	3.5	4.530
35.0	6.07	3.5	11.33
10.0	5.5	5.5	7.41
35.0	3.5	0.93	3.70
35.0	3.5	6.07	14.68
67.18	3.5	3.5	22.44
10.0	1.5	5.5	6.70
60.0	1.5	1.5	12.06
10.0	1.5	1.5	5.48
35.0	3.5	3.5	12.18
35.0	3.5	3.5	12.2
60.0	1.5	5.5	22.84
35.0	0.93	3.5	10.91
60.0	5.5	5.5	22.10

**Table 8:** Ranges and optimal concentrations of independent variables of the MCM obtained from the response surface model.

Independent Variable	Low, g/dm^3^	High, g/dm^3^	Optimum, g/dm^3^
Glucose	14.2562	65.7438	50.4581
Peptone	0.925623	6.07438	4.30301
Yeast extracts	0.925623	6.07438	4.30061

**Table 9:** Ranges and optimal concentrations of independent variables of MYB obtained from the response surface model.

Independent Variable	Low, g/dm^3^	High, g/dm^3^	Optimum, g/dm^3^
Glucose	2.82029	67.1797	67.1797
Yeast extract	0.925623	6.07438	1.24238
Malt extract	0.925623	6.07438	6.07438

**Table 10:** Kinetic parameters of *X. karsticola* growth models in optimized medium.

Medium	Logistic Curve Model			Reversible Autocatalytic Growth			
	µ_max_, d^−1^	β, g/dm^3^·d	R^2^	k_1_, d^−1^	$S_{0}^{\prime }$, g/dm^3^	K/1 + K	R^2^
MYB	0.711 ± 0.003 ^b^	0.0311 ± 0.0002 ^b^	0.9946	0.0363 ± 0.0012 ^a^	23.05 ± 0.08 ^a^	0.9900 ± 0.001 ^a^	0.9913
MCM	0.803 ± 0.004 ^a^	0.0389 ± 0.0002 ^a^	0.9963	0.0359 ± 0.0004 ^a^	21.27 ± 0.04 ^b^	0.9644 ± 0.005 ^b^	0.9868

Data are expressed as mean ± SD (n = 3); a, b: different letters in the rows indicate significantly different values (Tukey HSD tests, *p* < 0.05).

**Table 11:** Disk diffusion assay.

	Biomass Extracts							Culture Liquid Extracts				Controls (10 µg/disk)	
	BWE	BME	BEE	BBE	BHE	BEAE	BMCE	CLMCE	CLBE	CLHE	CLEAE	Gentamicin	Ketonazole
*Escherichia coli* ATCC 8739	-	-	-	-	-	-	9.67 ± 0.58	-	-	-	-	10 ± 0.00	-
*Eterococcus faecalis* ATCC 19433	-	-	-	-	-	-	-	-	-	-	-	15 ± 0.58	-
*Salmonella enterica* ssp. *enterica* ser. Enetritidis ATCC 13076	-	-	-	-	-	-	-	-	-	-	-	16 ± 0.58	-
*Listeria monocytogenes* ATCC 8787	-	-	-	-	-	-	8 ± 0.00	9 ± 0.58	-	-	-	23 ± 1.00	-
*Staphylococcus aureus* ATCC 25923	-	-	-	-	-	-	-	-	-	-	-	13 ± 0.00	-
*Pseudomonas aeruginosa* ATCC 9027	-	11 ± 1.00	-	15 ± 0.58	16 ± 1.00	20 ± 1.53	15 ± 0.58	-	-	-	8 ± 0.00	17 ± 0.58	-
*Proteus vulgaris* G	-	-	-	-	-	-	-	-	-	-	-	22 ± 0.58	-
*Klebsiella pneumoniae* ATCC 13883	-	-	-	-	-	-	-	-	-	-	-	13 ± 1.00	-
*Candida albicans* ATCC 10231	-	-	-	-	-	8 ± 0.00	8 ± 0.00	8 ± 0.00	9 ± 0.58	-	-	-	20 ± 0.58
*Bacillus subtilis* ATCC 6633	-	-	-	-	8 ± 0.00	9 ± 0.58			-	-	-	20 ± 1.00	-
*Bacillus cereus* ATCC 11778	-	-	-	-	-	-	9 ± 0.58	9 ± 1.00	-	-	11 ± 0.00	18 ± 1.00	-
*Wickerhamomyces* *anomalus*	8 ± 0.00	-	24 * ± 0.58	-	-	-	-	-	9 ± 1.00	-	-	-	28 ± 0.58
*Rhodotorula mucilaginosa*	-	-	-	-	-	-	-	-	-	-	-	-	34 ± 1.00
*Saccharomyces cerevisiae*	-	-	-	-	-	-	-	-	-	-	-	-	25 ± 0.58
*Saccharomycodes ludwigii*	10 ± 0.00	-	-	-	-	12 ± 1.00	-	-	-	12 ± 0.58	-	-	26 ± 1.00
*Pichia membranifaciens*	9 ± 0.58	10 ± 0.58	-	-	-	-	-	-	-	-	-	-	38 ± 1.00

Data are expressed as mean ± SD (n = 3); d_disk_ = 6 mm; “-”—no inhibition zone; *—single colonies present in the inhibition zone.

**Table 12:** Minimal inhibitory concentration of the *X. karsticola* extracts.

Test-Microorganism	Minimal Inhibitory Concentration, mg/cm^3^								
	BWE	BHE	BEAE	BMCE	CLBE	CLHE	CLEAE	Gentamicin	Ketonazole
*Escherichia coli* ATCC 8739	-	-	-	2.12	-	-	-	0.02	-
*Pseudomonas aeruginosa* ATCC 9027	-	0.067	0.59	-	-	-	-	0.0025	-
*Candida albicans* ATCC 10231	-	-	-	-	3.22	-	-	-	0.00063
*Bacillus subtilis* ATCC 6633	-	-	1.66	-	-	-	-	0.0025	-
*Bacillus cereus* ATCC 11778	-	-	-	-	-	-	6.25	0.0012	-
*Wickerhamomyces anomalus*	-	-	-	-	1.61	-	-	-	0.005
*Saccharomycodes ludwigii*	0.02	-	-	-	-	0.04	-	-	0.02
*Pichia membranifaciens*	0.05	-	-	-	-	-	-	-	0.0012

## Data Availability

Data is contained within the article.

## Abbreviations

The following abbreviations are used in this manuscript:

NBIMCC	National Bank of Industrial Microorganisms and Cell Cultures
MCM	Mushroom Complete Medium
MEB	Malt extract broth
MYB	Malt extract yeast extract broth
PDB	Potato dextrose broth
CDB	Czapek–Dox Broth
CCD	Central Composite Design
PTFE	Polytetrafluoroethylene
ATCC	The American Type Culture Collection
CFU	Colony-Forming Unit
CLSI	Clinical and Laboratory Standards Institute
SD	Standard deviation
Glu	Glucose
YE	Yeast extract
P	Peptone
MIC	Minimal inhibitory concentration
BWE	Biomass Water Extract
BME	Biomass Methanol Extract
BEE	Biomass Ethanol Extract
BBE	Biomass Buthanol Extract
BHE	Biomass Hexane Extract
BEAE	Biomass Ethyl Acetate Extract
BMCE	Biomass Methylene Chloride Extract
CLMCE	Cultural Liquid Methylene Chloride Extract
CLBE	Cultural Liquid Buthanol Extract
CLHE	Cultural Liquid Hexane Extract
CLEAE	Cultural Liquid Ethyl Acetate Extract
